# Effective Identification of Gram-Negative Bacterial Type III Secreted Effectors Using Position-Specific Residue Conservation Profiles

**DOI:** 10.1371/journal.pone.0084439

**Published:** 2013-12-31

**Authors:** Xiaojiao Yang, Yanzhi Guo, Jiesi Luo, Xuemei Pu, Menglong Li

**Affiliations:** College of Chemistry, Sichuan University, Chengdu, P.R.China; University of South Florida College of Medicine, United States of America

## Abstract

**Backgroud:**

Type III secretion systems (T3SSs) are central to the pathogenesis and specifically deliver their secreted substrates (type III secreted proteins, T3SPs) into host cells. Since T3SPs play a crucial role in pathogen-host interactions, identifying them is crucial to our understanding of the pathogenic mechanisms of T3SSs. This study reports a novel and effective method for identifying the distinctive residues which are conserved different from other SPs for T3SPs prediction. Moreover, the importance of several sequence features was evaluated and further, a promising prediction model was constructed.

**Results:**

Based on the conservation profiles constructed by a position-specific scoring matrix (PSSM), 52 distinctive residues were identified. To our knowledge, this is the first attempt to identify the distinct residues of T3SPs. Of the 52 distinct residues, the first 30 amino acid residues are all included, which is consistent with previous studies reporting that the secretion signal generally occurs within the first 30 residue positions. However, the remaining 22 positions span residues 30–100 were also proven by our method to contain important signal information for T3SP secretion because the translocation of many effectors also depends on the chaperone-binding residues that follow the secretion signal. For further feature optimisation and compression, permutation importance analysis was conducted to select 62 optimal sequence features. A prediction model across 16 species was developed using random forest to classify T3SPs and non-T3 SPs, with high receiver operating curve of 0.93 in the 10-fold cross validation and an accuracy of 94.29% for the test set. Moreover, when performing on a common independent dataset, the results demonstrate that our method outperforms all the others published to date. Finally, the novel, experimentally confirmed T3 effectors were used to further demonstrate the model’s correct application. The model and all data used in this paper are freely available at http://cic.scu.edu.cn/bioinformatics/T3SPs.zip.

## Introduction

Currently, eight types (type I to type VIII) of secretion systems in Gram-negative bacteria have been identified according to their outer membrane secretion mechanisms [1]. With these systems, various types of proteins and virulence factors are secreted and transported to the surrounding environment to promote bacterial survival [2]. The type III secretion system (T3SS) is a specialised protein delivery system that has been widely encoded in many Gram-negative bacteria, such as *Salmonella, Yersinia, Shigella, Escherichia, Pseudomonas, etc.* [3], [4]. T3SSs are central to the pathogenesis and virulence of many important Gram-negative bacterial pathogens. Bacterial type III secreted proteins (T3SPs) are delivered into host cells specifically via T3SSs, which play important roles in host-pathogen interactions and have proven to be powerful weapons used by pathogens against the host immune defenses [4], [5]. Because the secretion mechanism of T3SS is not yet fully understood, the recognition of novel T3SPs is an important and challenging task for the study of T3SSs. However, to adapt in different hosts and defend against immune system attacks, T3SPs evolve very quickly and their amino acid sequences are highly variable [6]. Many T3SPs have no homology with other effectors in public databases [7]. Therefore, it is notoriously challenging to develop a reliable computational method to effectively identify T3SPs.

In recent years, many computational methods have been proposed to predict the functions of secreted proteins [8]–[13]. Due to the great diversity and feature specificity of T3SP sequences, these methods cannot be simply repurposed to predict T3SPs. Thus, special approaches have been developed to predict T3SPs. Methods based on sequence alignment were originally proposed [14]–[16], but the low sequence similarity between different T3SPs leads to the poor performance of these methods.

We know that secretion is the process that the secreted proteins are produced from the bodies of bacterial and translocation refers to translocate the effectors from the bacterial cytosol into host cells. So T3SPs typically include a “leader sequence” that signals export, a chaperone-binding sequence or translocation signals following the leader sequences [17]–[19]. It has been reported that the actual secretion signal is generally in the first 30 amino acids [20]–[25], but the secretion of T3SPs needs to be guided by the chaperone-binding sequences or translocation signals [18], [19], [26]–[28]. Studies have also detected an amino acid composition (AAC) bias in the N-terminal sequences of T3SPs. For example, Guttman *et al.* found high Ser content and the low Asp content in the first 50 amino acids of most effectors in *P. syringae* [29]. Based on the general features of the N-terminal peptides, different machine learning methods have been adopted to predict T3SPs, e.g., Naive Bayes (NB) [30], artificial neural network (ANN) [31] and support vector machine (SVM) [27], [29], [32]–[35]. Arnold *et al.* [30] developed the first universal *in silico* program, EffectiveT3, by incorporating the AAC and secondary structures of N-terminal residues. Löwer and Schneider [31] proposed an ANN model based on a sliding window to capture signal features among the first 30 amino acids. This model gives high selectivity but low sensitivity. Sato *et al*. [32] introduced N-terminal flexibility, structure-related parameters and a codon adaptation index to refine the discriminatory power of the classifier. With the AAC features, secondary structure and accessibility information, a SVM model by Yang *et al.* [33] achieves a sensitivity of 65% for *P. syringae* T3SPs. However, except the method by Samudrala, et al. [27], none of these methods utilise position-specific features. Most recently, Wang *et al.* developed a BPBAac model based on position-specific AAC components for T3SP prediction. This model gives very high sensitivity of 97.42% in classifying T3SPs and non-T3 proteins [34]. Dong *et al*. used the profile-based k-spaced amino acid pair composition (HHCKSAAP) to represent the N-terminal sequences and they called this new method, BEAN [35]. This method has been demonstrated to be superior to others. However, the position-specific residues containing important signal information remain to be identified. Moreover, these methods are all developed for classifying T3SPs and non-T3 proteins. Since previous studies have successfully identified the secreted proteins (SPs) from other proteins [8]–[11], [36], it is more essential, but also more difficult to distinguish T3SPs from other SPs (non-T3 SPs). Moreover, the confirmed T3SPs used by previous studies are not sufficient. The largest number of T3SPs was 595, which was used by Löwer and Schneider [31]. However, most were not validated, and some with high homologies were not excluded, which affects the generalisability of the predictions.

In this work, experimentally verified T3SPs from 16 bacterial species were collected, and the non-T3 SPs from seven other types (T1, T2 and T4–T8) were all included. Thus, a comprehensive and unbiased dataset with lower than 25% sequence similarity was constructed, and a new method based on position-specific residue conservation profiles was proposed to identify T3SPs from the non-T3 SPs.

As reported, the leader sequences are commonly encoded within the first 30 residues [20]–[25], [37], but it is difficult to develop heuristic rules to identify them [27], [30]. The translocation of many effectors also depends on the chaperone-binding domains or translocation signals [18], [19], [26] that span residues 25 to 100 [18]. However, among the 100 residues, those that are distinctive for T3SPs prediction have not been identified. In this paper, we make a first attempt to identify them. First, the conservation profiles of the N-terminal 100 residues were constructed using a position-specific scoring matrix (PSSM). Then taking the PSSM profiles of residues as the inputs, significance analysis was implemented to reveal the significant differences between T3SPs and non-T3 SPs using SAM. Thus, 52 functionally important residues were identified, including all the first 30 amino acid residues that are the signal peptide and the 22 chaperone-binding or translocation residues beyond the first 30 positions.

Using the protein sequence information, including AAC, solvent accessibility information, secondary structure and six physicochemical properties, the sequence fragment of 52 position-specific residues was translated into 250 numerical variables. Permutation importance analysis was used to optimise these features, and 62 optimal features were selected. Finally, a prediction model was developed using random forests (RF). The model gives a high receiver operating curve (AUC) of 0.9277 for the 10-fold cross validation and an accuracy of 92.56% for the test set. Moreover, when constructing a common independent dataset, the results demonstrate that this method is superior to the existing sequence-based methods. To demonstrate the model’s application, new experimentally verified effectors were predicted by our model, and nearly all sequences were successfully identified.

## Materials and Methods

### Dataset Collection

Although the in-depth study of bacterial secretomes has progressed and an increasing number of secreted proteins have been discovered from Gram-negative bacteria, the sequence data have not yet been systematically integrated. To collect the secreted proteins, an extensive literature search was carried out to obtain information on protein or gene IDs and certain secretory types. Then, the corresponding sequences were collected from Swiss-Prot and TrEMBL [38]. After eliminating sequences with less than 100 amino acids, 1139 SPs were collected, including all secretory types from type I to type VIII. This dataset consists of 787 non-T3 SPs and 352 T3SPs. In addition, other experimentally verified T3SPs were also collected from the previous works of Wang *et al.* [34] and Tay *et al.* [39], respectively, to increase the comprehensiveness of the T3SP data. In total, the dataset of T3SPs contains 662 protein sequences. According to the different secretion mechanisms, the proteins are grouped into eight classes and shown in Supplementary Table S1.

Reports have shown that the translocation of some effectors only depends on the first ∼50 residues, e.g. AvrBs2 in *Xanthomonas* [19], Tir in *Enteropathogenic Escherichia coli* [40] and PopD in *Pseudomonas aeruginosa* [41]. Moreover, the conserved chaperone-binding domain found by Costa *et al.* only covers the first 25 to 45 N-terminal residues [28]. But there are still many other effectors that require the first ∼100 residues to be secreted or trans-located. For example, translocation signals in some Yops are located in the first 50–100 residues [42], [43]. The chaperone-binding site of SopE covers the first 39 to 77 N-terminal residues [44] and that of YopE is located between amino acid residues 15–100 [45]. The chaperone SycH binding domain in YopH extends from residues 20 to 70 [46]. It also has been proven that the region between residues 15 and 78 of SopE is responsible for binding the chaperone InvB [47]. Moreover, previous studies have shown that the maximal secretion or translocation may require the first 100 amino acids [7], [19], [33], [36], [48], in which the signal peptides of T3SPs may be contained [34], [49]–[51]. Instead of using full-length protein sequences for prediction, we only focused on the N-terminus in this study. Thus, only the first 100 residues were extracted from the dataset in all following calculations.

Further, to avoid homology bias, a strict criterion was used to evaluate our method, such that the mutual identity in the dataset was less than 25% [52]–[55]. Firstly, the non-redundant dataset was obtained by clustering the sequence segments of 100 residues with CD-HIT [56] at 30% identity threshold. Then another common tool, BLASTCLUST program [57] with parameters “-S 25 -L 0.9 -b F” was used to ensure that no clear homologues are present in the non-redundant dataset. In this way, any two protein segments from different clusters shared less than 25% identical residues over 90% coverage of any protein. Finally, an unbiased dataset that included 283 T3 and 313 non-T3 proteins was constructed.

### Position-specific Conservation Profiles and Significance Analysis

The first 100 amino acids have been shown to be very important for the secretion of T3SPs, but those that are distinctive for T3SPs prediction remain to be identified. One fact is that the conservation varies from site to site and some residues with little conservation have no contributions to T3SPs identification. A position-specific scoring matrix (PSSM) has been considered as an effective measure of residue conservation in a given location. It has been widely used to predict the functional sites [58]–[63] and received good results. Studies have shown that evolutionary profiles obtained from multiple sequence alignment contain more comprehensive information than a single sequence [64], [65]. Here, we used PSSM to construct the conservation profiles of the first 100 residues using PSI-BLAST to search the Swiss-Prot for multiple sequence alignment against the query protein. A conservation profile of a residue consists of the log-likelihoods of the mutation of 20 standard amino acids. In this way, a protein was represented into a matrix of 100*20.

To identify the distinct position-specific residues for T3SPs prediction, a significance analysis was performed to extract the conservation differences of the 100 residues between T3SPs and non-T3 SPs using significance analysis of microarrays, (SAM) established by Tusher *et al.* [66]. SAM is a statistical technique for identifying significant genes and is one of the most popular methods employed for microarray analysis [67]–[69]. Similar to T-test, SAM depends on constructing a test statistic to estimate the null distribution. To identify significant genes, a false discovery rate (FDR) is proposed as the expected proportion of false positive findings among those differential expressions [66], [67]. FDR is commonly presented as a q-value, which is the minimum FDR. A q-value threshold is used to control the FDR at a desirable level. For selecting significant genes, the input is the expression value of a gene. Here, SAM was used to detect the significant residues, so the input is the PSSM profile of a residue. The smaller the q-value is, the more significant the conservation difference is. A q-value equalling to 0 means a significant difference with a 100% confidence level. In our paper, a q-value threshold of 0.05 was used to determine whether the conservation profiles of the two residues at the same position are remarkably different between T3SPs and non-T3 SPs. We performed SAM analysis using the *siggenes* package in *R* language (version 2.13.1) by the following link: http://www.bioconductor.org/packages/release/bioc/html/siggenes.html.

### Feature Space

After reviewing the previous methods for predicting T3SPs, four protein features were commonly used in the learning model, including amino acid composition (AAC) [7], [29], [33], secondary structure (SS) [7], [30], [33], relative solvent accessibility (RSA) [7], [33] and physicochemical properties (PP) [32], [33], which are theoretical pI, total number of negatively charged residues of Asp and Glu (Nnc) and positively charged residues of Arg and Lys (Npc), instability index (Ins), aliphatic index (Ali) and grand average hydrophobicity (Hydro), respectively. The RSA and SS were predicted by the SABLE protein structure prediction server at http://sable.cchmc.org/ [70]–[72]. Using SABLE, the RSA and contents of three SS types (helix, beta strand and coil) were obtained for each residue. Here, helix, beta strand and coil are represented as SSH, SSE and SSC, respectively. Six physicochemical properties were estimated for the N-terminal sequence by the Protparam program at http://web.expasy.org/protparam/ [73].

### Random Forest and Permutation Importance Analysis

The random forest (RF) algorithm proposed by Breiman [74] has been successfully applied to dealing with various biological problems, such as miRNA–target interactions [75]–[77]. With the advantage of bootstrap aggregating (bagging) [78], RF is especially powerful for the data sets with a large number of features [79]. A group of decision trees in bagging utilised by RF can reduce the variance of single trees and thus improve the prediction accuracy. Moreover, out-of-bag (OOB) data are used to estimate the test error of a RF model. Given a training set, two thirds of the samples (in-the-bag) are randomly selected. The remaining samples, called the OOB data, are left out. In this way, a RF model gives a good tolerance for the noisy data. According to Breiman’s description [74], *n* decision trees (*ntree*) are trained on different bootstrap samples from the training data. Each tree is fully grown and left unpruned. Instead of using all features, RF splits each node by the best split among randomly sampled *m* predictors (*mtry*) at that node when growing a tree. In this work, the two parameters, *ntree* (the number of trees to grow) and *mtry* (the number of variables randomly selected as candidates at each node), were optimised using a grid search approach; the value of *ntree* was from 500 to 2500 with a step length of 500, and the value of *mtry* was from 1 to 40 with a step length of 1.

Meanwhile, RF provides two feature importance measures, including Variable Importance (VI) and Gini Importance (GI) [78], [79]. The VI value of one feature is computed according to the average decrease of the model accuracy on the OOB samples when this feature is randomly permuted. Thus, it is also called permutation accuracy importance measure. The GI is related to the impurity of the split of a node because some features may be redundant or have little relevance to T3SPs prediction. To distinguish significant features from uninformative ones and to further avoid overfitting, the permutation importance measure was adopted to evaluate each feature’s contribution to the classifier in our work. The RF algorithm was implemented by the RF package in *R*.

### Performance Evaluation

For two-class classification problems, four parameters, sensitivity (Sn), specificity (Sp), accuracy (Acc) and Matthews Correlation Coefficient (MCC) were utilised to evaluate the classifier performance. They are defined as follows:
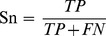
(1)

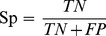
(2)

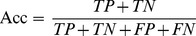
(3)

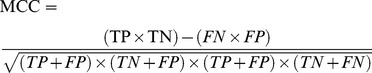
(4)Where TP, TN, FP and FN represent true positives, true negatives, false positives and false negatives, respectively.

To further evaluate the performance of our method, Receiver Operating Characteristic (ROC) curves and the areas under ROC curves (AUC) [80] were utilised. The maximum value of the AUC is 1.0, which denotes a perfect prediction. A random guess gives an AUC value of 0.5.

## Results and Discussion

### Distinct Position-specific Residues for T3SPs

**Figure 1:**
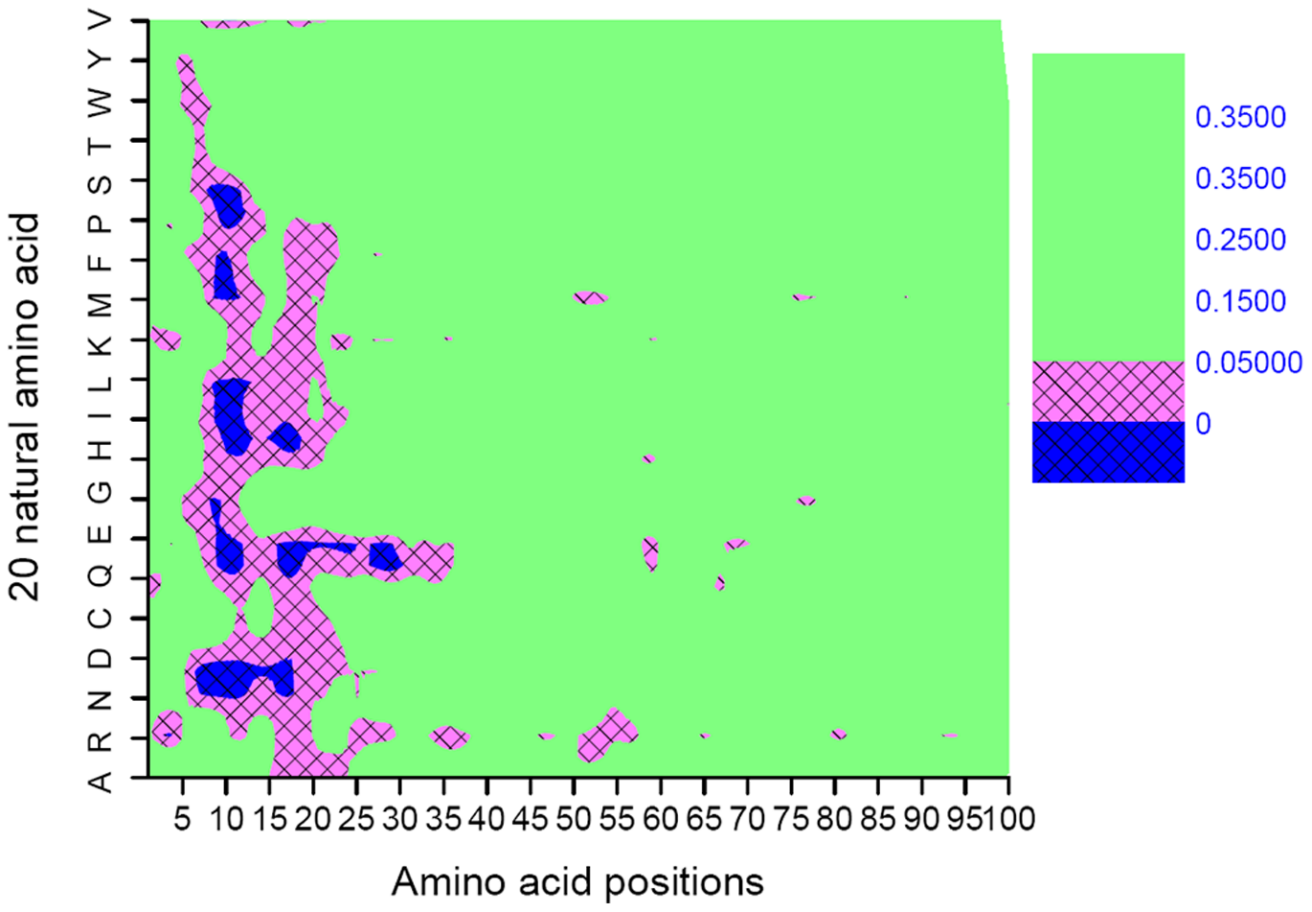
Significant difference analysis by SAM for the first 100 residues. The positions with q-values less than 0.05 are in pink, including the blue parts with q equaling to 0. Those with q> = 0.05 are in green.

The PSSM profiles were calculated for the whole sequences of 283 T3SPs and 313 non-T3 SPs and those of the N-terminal 100 residues were retrieved. Each residue was characterised by 20 values representing the log-likelihoods of mutation of the 20 standard amino acids. Thus, for each position, two matrices were built, including a T3SP matrix of 20*283 (matrix A) and a non-T3 SP matrix of 20*313 (matrix B). Then, SAM was used to implement significant difference analysis on matrix A and B. Finally, 20 q-values were obtained, representing the difference scores of the 20 log-likelihoods of mutation of the 20 standard amino acids. The analysis results by SAM for all 100 residues are shown in Figure 1. The pink parts represent the positions with q-values less than 0.05, indicating that significant differences are detected by SAM at these positions between T3SPs and non-T3 SPs. We can see that most q-values of less than 0.05 are concentrated at the first 30 residue positions and that all blue parts with q-values equal to 0 are also distributed at this region, indicating that the signal information is mainly contained among the first 30 amino acids. However, there are still other scattered positions (pink parts) that contain a significant conservation difference beyond the first 30 residues.

To quantitatively characterise the difference between T3SPs and non-T3 SPs at these 100 positions, if m of the 20 q-values at each position is less than the threshold of 0.05, the ratio of the significant difference (RSD) is defined according to the following equation:
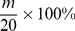
(5)


**Figure 2:**
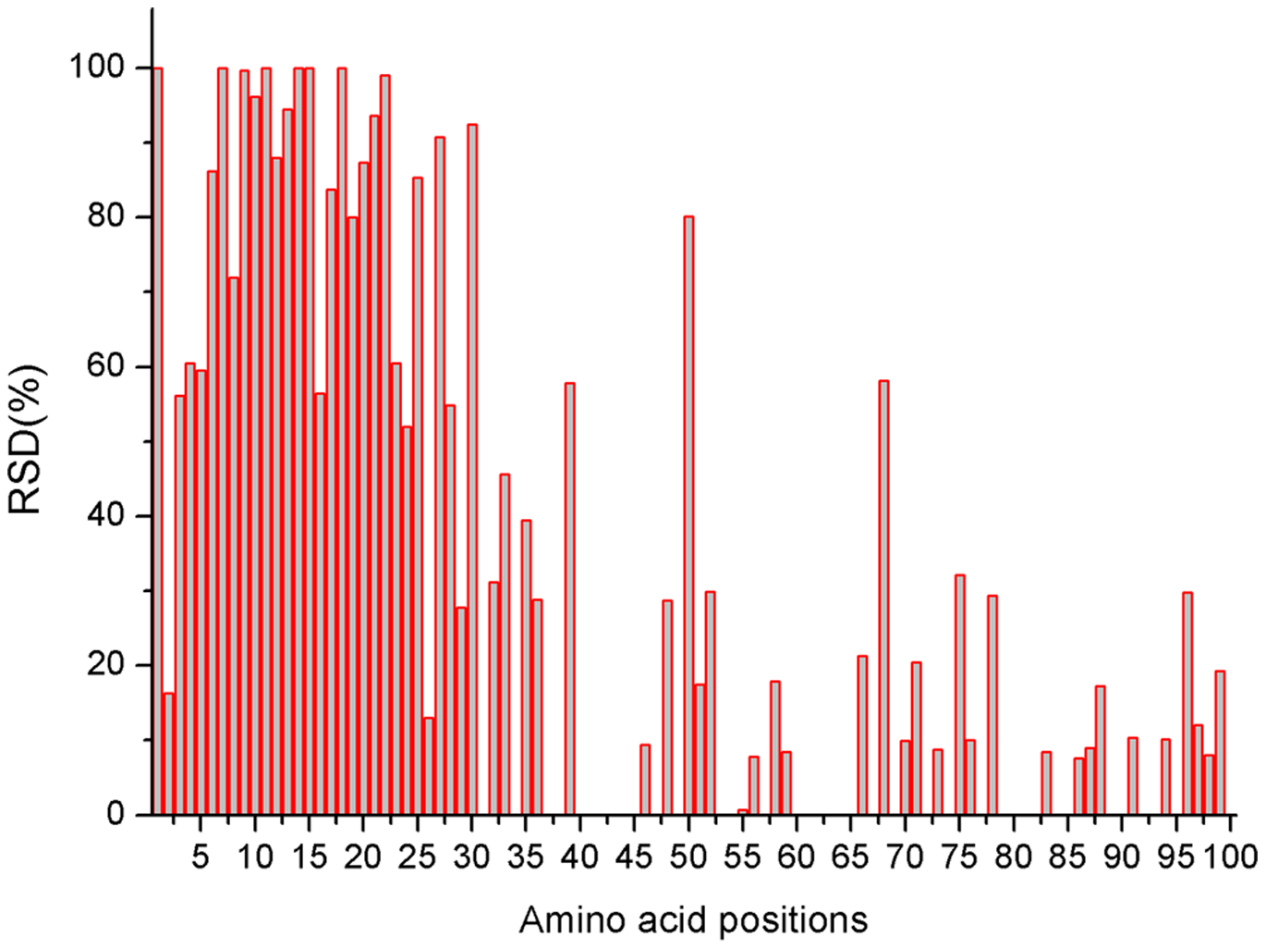
The bar charts of the RSDs for the 100 residues positions. The higher RSD value of one residue position means the more significant conservation difference between T3SPs and non-T3 SPs at this position. The positions with RSD values of <10% are not distinctive residues.

The RSDs for the 100 residues are shown in Figure 2, and the detailed values are listed in Table 1. We find that there are 38 residues with no significant conservation difference between T3SPs and non-T3 SPs. The 20 q-values of each residue are all higher than 0.05 and have an RSD value of 0. However, the RSDs of the remaining 62 residues are higher than 0, indicating that the conservation differences exist between T3SPs and non-T3 SPs at these positions. They are most likely the distinct position-specific residues for T3SPs.

**Table 1:** The conservation differences analyzed by SAM for N-terminal 100 residues.

RSD (%)	Total number of residues	Corresponding positions
100	6	1 7 11 14 15 18
90∼100	7	9 10 13 21 22 27 30
80∼90	7	6 12 17 19 20 25 50
70∼80	1	8
60∼70	2	4 23
50∼60	7	3 5 16 24 28 39 68
40∼50	1	33
30∼40	3	32 35 75
20∼30	8	29 36 48 52 66 71 78 96
10∼20	10	2 26 51 58 76 88 91 94 97 99
0∼10	10	46 55 56 59 70 73 83 86 87 98
0	38	31 34 37 38 40 41 42 43 44 45 47 49 53 54 57 60 61
		62 63 64 65 67 69 72 74 77 79 80 81 82 84 85 89 90
		92 93 95 100

**Figure 3:**
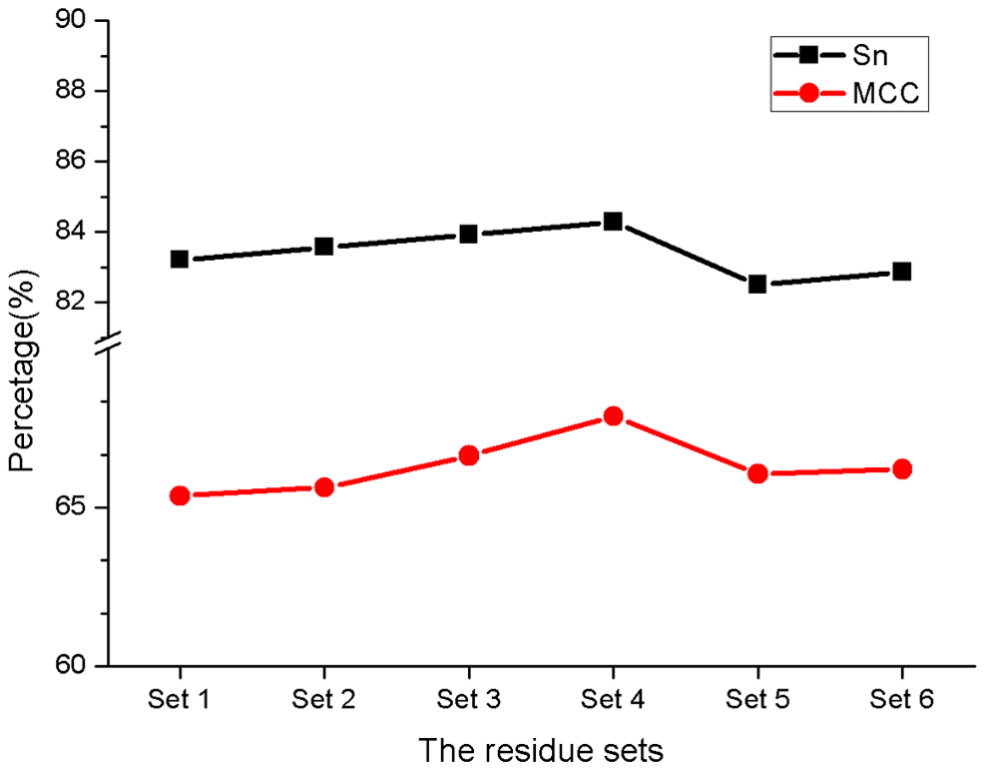
The prediction results of models based on the six residue sets respectively. Set 4 includes all 52 distinct residues and the model based only Set 4 yields the highest Sn and MCC.

To confirm these distinct residues, according to the RSD values of the 100 residues, six residue sets were selected for the following feature representation and model training. Set 1 includes 20 residues with RSD>80%. Set 2 includes 31 residues with RSD>40%. Set 3 includes 42 residues with RSD>20%. Set 4 includes 52 residues with RSD>10%. Set 5 includes 62 residues with RSD>0, and Set 6 includes all 100 residues. Here, for selecting distinct residues, only residues’ descriptors of RSA and SS were used for the representation of each residue. So the six residue sets (Set 1 to 6) contain 80, 124, 168, 208, 248 and 400 feature variables, respectively. Finally, the positive and negative datasets were randomly divided into a training set and test set by a 9∶1 ratio, respectively, and the process of random selection was repeated 10 times. Thus, six RF models were trained and tested. The comparison results are shown in Figure 3, and the detailed information can be observed in Table 2. We can see that the RF model based on Set 4 gives the highest sensitivity and MCC, which are 84.29% and 0.68, respectively. Set 4 includes 52 residues with RSD >10%. It is noticeable that if at least two q-values of a given residue is less than 0.05, we can infer it is a distinct residue for T3SPs. It also indicates that SAM is very sensitive in detecting the conservation difference between T3SPs and non-T3 SPs and further proves that for a given protein, the function of its residues is always conservative in the evolutionary process.

**Table 2:** The performance of the RF models based on the six residue sets.

Residue sets	RSD (%)	No. of residues	No. of variables	Sn (%)	Sp (%)	Acc (%)	MCC
1	>80	20	80	83.21±7.13	81.94±6.67	82.54±4.86	0.6536±0.09
2	>40	31	124	83.57±6.52	81.94±8.04	82.71±6.15	0.6563±0.12
3	>20	42	168	83.93±6.37	82.58±4.85	83.22±4.73	0.6663±0.08
4	>10	52	208	84.29±6.78	83.23±6.77	83.73±3.12	0.6787±0.06
5	>0	62	248	82.50±7.61	83.23±6.77	82.88±4.55	0.6606±0.09
6	≥0	100	400	82.86±7.94	82.90±6.45	82.88±8.37	0.6620±0.04

The first 30 residues are all exactly included in the 52 distinctive residues identified by our method (Table 1). Moreover, of these 30 positions, there are 27 residues with RSDs of higher than 50% (Figure 2). Thus, these data verify the conclusion that the first 30 N-terminal residue positions are most informative for T3SP prediction [31], [33]. In other words, the leader sequence of a T3SP can be successfully identified by our method. Meanwhile, we also identified 22 additional distinct residues that are in the chaperone-binding region or translocation signals, and they also contain important signal information for T3SP secretion. These findings fit well with the reports that the T3SP secretion needs to be guided by the chaperone-binding sequences or translocation signals [18], [26]–[28].

### Permutation Importance Analysis by RF

Previous studies have examined four protein feature characteristics, i.e., AAC, SS, RSA and PPs, that may contribute to the mechanisms underlying T3SP secretion [29], [31]–[33]. Based on the 52 distinct residues, each protein sequence was translated into a vector of 234 numerical descriptors by combining these features (52×4+20+6). However, the contribution of individual feature variables has not been measured, and no conclusive remarks can be drawn. In addition, the number of variables (234) is relatively larger than the number of T3SPs (283), which would lead to overfitting. For feature compression and optimisation, permutation importance analysis was adopted as a criterion for measuring the contribution of individual features to T3SP prediction. Nine-tenths of the samples were randomly selected from T3SP and non-T3 SP datasets for feature importance scoring. The process was repeated 100 times with random re-sampling of the constructed models, and the feature measure scores were averaged.

**Figure 4:**
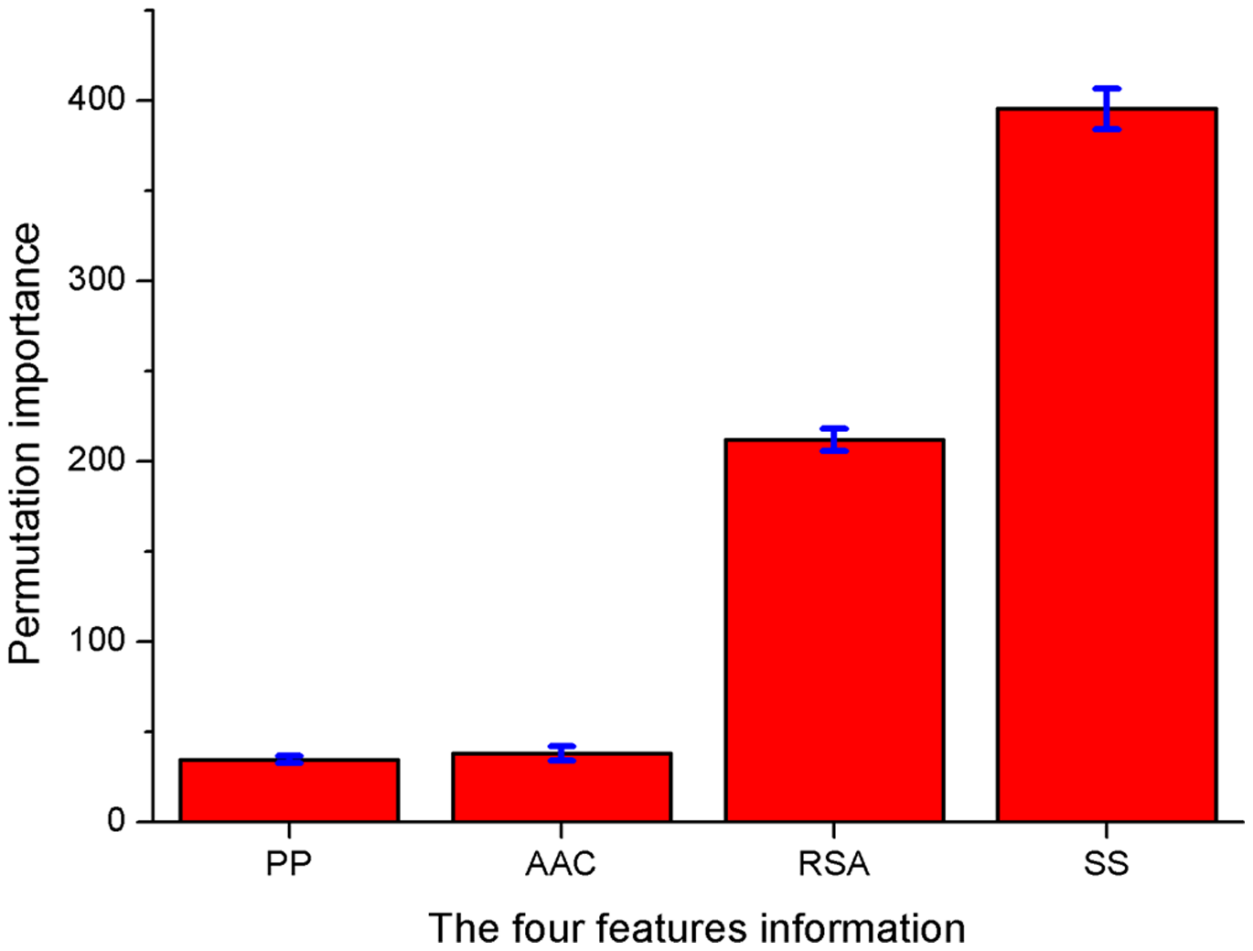
Feature importance measure for AAC, SS, RSA and PP respectively. AAC, SS, RSA and PP denote amino acid composition, secondary structure, relative solvent accessibility and physicochemical properties, respectively.

To evaluate the contributions of the four features AAC, SS, RSA and PP, the total importance score of each feature was calculated (Figure 4). Figure 4 shows that SS and RSA give the highest feature importance scores, which means that both SS and RSA are important for T3SP prediction. This finding is consistent with the observations that neither SS nor RSA could improve classification performance [30] and that the combination of SS and RSA could improve model performance [33].

Though AAC and PP are less important, with relatively low importance scores, some individual variables in them are still important. The importance scores of the 234 variables are given in descending order (Supplementary Table S2). To select the optimal variables, according to the coverage ratio of each variable, five feature groups (marked G1 to G6) were built, and the sum of coverage ratio was 90%, 80%, 70%, 60%, 50% and 40%, including 139, 106, 81, 62, 47 and 34 variables, respectively. It shows that except for the top 139 variables, the remaining 95 variables only have a coverage ratio of 10%, which indicates that some redundancy does exist in the whole feature set.

**Figure 5:**
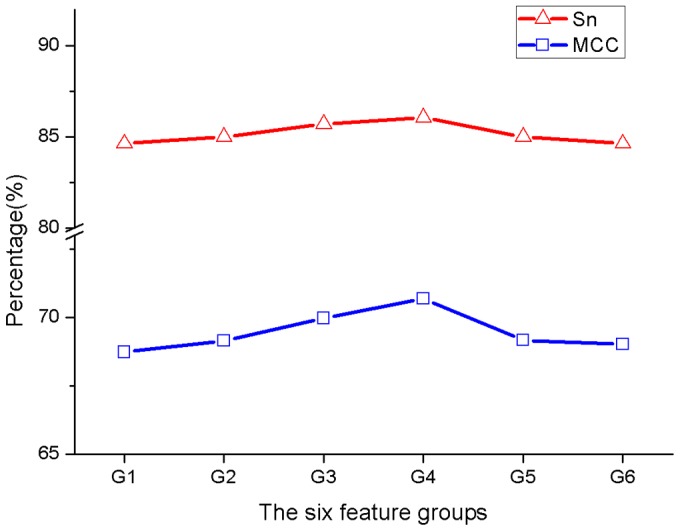
The prediction results of the models based on five feature groups respectively. The feature group G4 contains 62 individual variables and the model of G4 gives the best performance, so they are selected as the optimal feature set.

Thus, for further feature optimisation, six feature groups were trained and tested by RF. The performance results are shown in Figure 5 and Table 3. From Figure 5, we can see that the RF predictor based on G4 (62 variables) gives the highest Sn and MCC. Thus, in the following experiments, these optimal 62 variables were selected for constructing the prediction model.

**Table 3:** The performance of the RF models based on the six feature groups.

Feature group	Coverage ratio (%)	No. of features	Sn (%)	Sp (%)	Acc (%)	MCC
G1	90	139	84.64±5.62	83.87±6.97	84.24±4.28	0.6873±0.08
G2	80	106	85.00±9.34	83.87±6.08	84.41±5.58	0.6915±0.11
G3	70	81	85.71±4.45	84.19±6.66	84.92±3.93	0.6997±0.07
G4	60	62	86.07±4.89	84.52±7.10	85.25±5.06	0.7069±0.10
G5	50	47	85.00±7.86	83.87±7.45	84.41±5.52	0.6917±0.11
G6	40	34	84.64±5.34	83.87±9.05	84.24±3.85	0.6902±0.07

**Figure 6:**
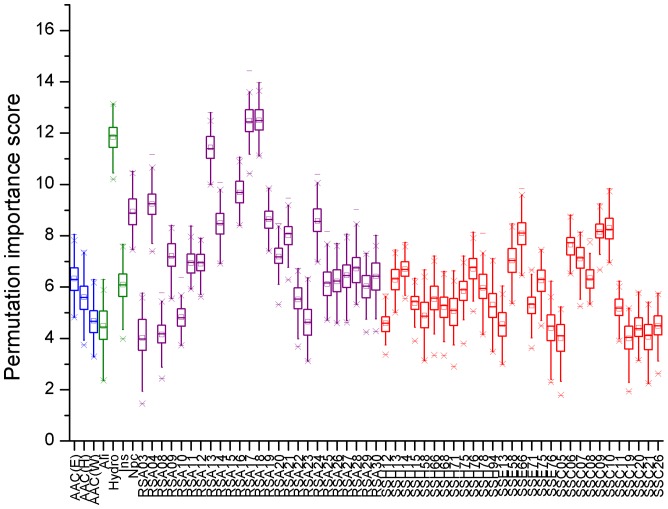
The importance score distributions of the 62 optimal individual variables. The blue, green, purple and red colors denote the four features of AAC, SS, RSA and PP respectively. AAC (E), AAC (H) and AAC (Y) are the amino acids composition of Glu, His and Trp respectively. Hydro, Ins, Npc and Ali represent hydrophobicity, instability index, the number of positively charged residues and aliphatic index, respectively. RSA is relative solvent accessibility. SSH, SSE and SSC are the three secondary structure types of helix, beta strand and coil respectively. The following number of RSA, SSH, SSE and SSC denotes the corresponding residue position.

The score distributions of these 62 optimal features are shown in Figure 6 and Table 4 lists their detailed information. We observed that among the 62 variables, an overwhelming number of them (55 variables) are still from SS and RSA. Obviously, a protein’s function and structure are intimately linked. This result well demonstrates that the structural features (SA and SS) are important for the secretion of effectors. It is interesting that most of those 55 variables are from the first 30 amino acids. All the 25 RSA variables are from the first 30 residues. From the 30 SS variables selected at this stage, there are 11 coil variables (SSC), which are all from the first 30 residues, and 10 of them distribute in the first 25 amino acids. This result exactly confirmed the observation drawn by Arnold *et al.* that coiled regions are enriched in the N-termini of T3SPs by counting the structural features (coil, helix, beta strand) at each residue within the first 25 amino acids [30]. In addition, our method also selected 12 helix and 7 beta strand variables (SSH and SSE), but most of them are from the residues beyond the first 30 positions. Thus, these results again demonstrate that the first N-terminal 30 residue positions are most informative for T3SPs prediction, but other positions within the 100 residues also contain important signal information for T3SP secretion.

**Table 4:** Statistic for the 62 optimal features selected by random forest.

The feature information		Number of variables	Corresponding variables
Amino Acid Composition		3	Glu, His, Trp
Physicochemical properties		4	Hydrophobicity
			Total number of positively charged residues
			Instability index
			Aliphatic index
Relative Solvent Accessibility		25	RSA (3, 4, 8∼30)
Secondary structures	helix	12	SSH (12∼15, 58, 66, 68, 71, 75, 76, 78, 94)
	beta strand	7	SSE (12, 13, 58, 66, 71, 75, 76)
	coil	11	SSC (5∼11, 19∼21, 26)

In addition, four PP variables were incorporated, including Hydro, Ins, Npc and Ali. Hydro has the third highest score among the 62 optimal variables. This result fits well with previous studies showing that T3SPs are likely to be unstable and hydrophobic [15], [30], [32]. For the AAC information, three AAC variables (Glu, His, Trp) were selected. Guttman *et al.* [29] have found a high proportion of Ser and a low proportion of Asp in T3SPs, but both residues were not included. The reason may be that this AAC bias is only for *P. syringae* effectors, and this is not accurate because other effectors do not possess this feature, as pointed by Yang [81]. In our dataset, a broad dataset of genomes from 16 organisms was collected, including *Aeromonas*, *Citrobacter rodentium*, *Edwardsiella tarda*, *Escherichia coli* and *Ralstonia solanacearum*. However, the research of Yahara has proved that the AAC of His is an important feature that directly contributes to the discrimination of effectors [82], which provides supports for our results.

### Performance Comparison with Current Prediction Models

We compared our method with four published methods for T3SPs prediction, Effective T3 [30], ANN based method [31], BPBAac [34] and BEAN [35]. In particular, BPBAac and BEAN are the more recently published method and report the highest prediction accuracy for T3SPs. Moreover, the data used in BEAN are all from those in BPBAac. To make a fair comparison among different models, a common independent dataset was achieved by comparing our dataset with those of Effective T3, ANN, BPBAac and BEAN. And then 92 T3 proteins were extracted, but they are not included in all four models. Because almost all negative samples were not different among our method and the three models, we only randomly retrieved 100 non-T3 SPs from our dataset for testing. Thus, in our prediction model, 191 T3SPs and 213 non-T3 SPs were remained as the training data. These 192 test proteins were predicted by BPBAac tool and the web-servers of Effective T3, ANN and BEAN, respectively. The prediction results are listed in Table 5. It clearly demonstrates that our method outperforms these three models. Although Effective T3 and BPBAac also yield a very high Sp of 97% and 92% respectively, the Sn is less than 30%. The Sn and Sp obtained by our model are 68.48% and 98.00%, respectively, which are significantly higher than those by ANN and BEAN. In addition, when conducting comparisons using our method, Effective T3, BPBAac and BEAN, more independent sequences (149 T3SPs) can be extracted. After retrieving the 160 non-T3 SPs, only a half of the data were used for training in our model, including 134 T3SPs and 153 non-T3 SPs. A comparison of the results of our method, Effective T3, BPBAac and BEAN on the 309 common independent sequences is shown in Table 6. It shows that our method still performs much better than Effective T3, BPBAac and BEAN. Our method gives a highest Sn of 71.14%. It also indicates that our model was also robust when a small-size training dataset was used.

**Table 5:** The performance of different models on the common independent proteins.

Model	Sn (%)	Sp (%)	Acc (%)	MCC
RF (Our method)	68.48 (63/92)	98.00 (98/100)	83.85(161/192)	0.7018
BPBAac	13.04 (12/92)	97.00 (97/100)	56.77 (109/192)	0.1870
EffectiveT3	26.09 (24/92)	92.00 (92/100)	60.42 (116/192)	0.2425
ANN	47.83 (44/92)	90.00 (90/100)	69.79 (134/192)	0.4203
BEAN	46.74 (43/92)	81.00 (81/100)	64.58(124/192)	0.2964

**Table 6:** Comparison results of our method, Effective T3, BPBAac and BEAN.

Model	Sn (%)	Sp (%)	Acc (%)	MCC
Our method	71.14	98.13	85.11	0.7406
BPBAac	16.78	98.75	59.22	0.2748
EffectiveT3	24.16	95.63	61.17	0.2857
BEAN	54.36	77.50	66.34	0.3282

Overall, compared to other four methods, our method gives the best performance. After a deep investigation, we can find that these methods are all based on all the residues at N-terminal region, but they ignore one fact that some residues with little conservation have no contributions to T3SPs identification. So some redundant feature information may be introduced by these non-distinct residues and weaken the predictive power. Our predictor was constructed only based on the distinct residues and then a permutation importance analysis was implemented to ensure a more optimal feature set, so the performance of our method was further improved.

### Predictor Construction and Testing

**Figure 7:**
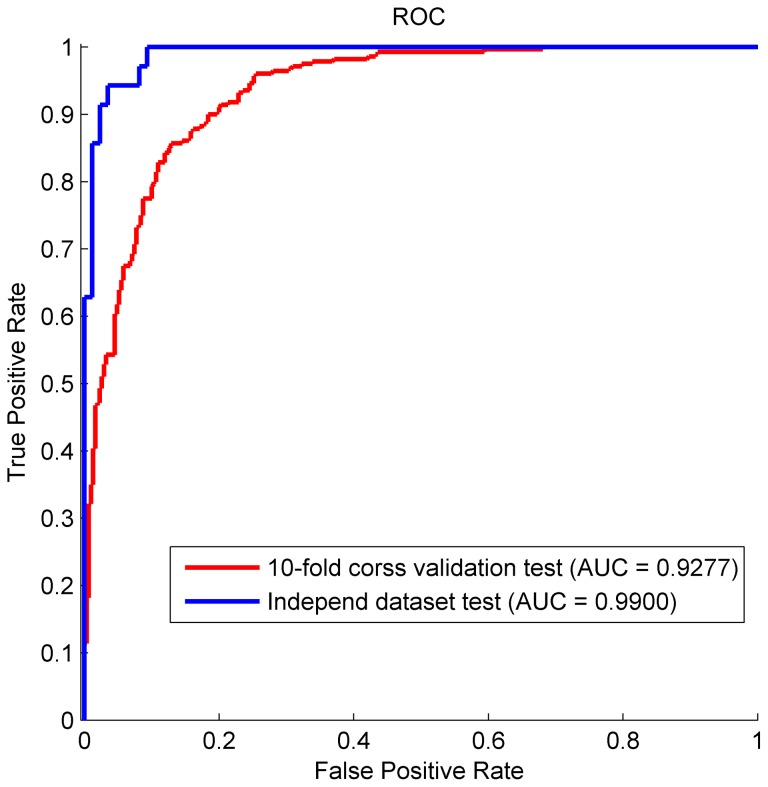
The ROC curves of 10-fold cross-validation test and the independent dataset test respectivley. The model gives a high receiver operating curve (AUC) of 0.9277 for the training set and 0.9900 for the independent test set.

Using the whole unbiased dataset of 283 T3SPs and 313 non-T3 SPs, the final RF predictor was constructed by 10-fold cross-validation. All samples were randomly divided into 10 subsets of similar size, 9 subsets were selected to train the model and the remaining ones were used to test the model. As shown in Table 7 and Figure 7, our model performed well. The average Sn, Sp and Acc are 86.07%, 84.52%, 85.25%, respectively. According to the ROC curve, the method yields a high AUC of 0.9277.

**Table 7:** The performance of the RF model by 10-fold cross validation test.

Test	Sn (%)	Sp (%)	Acc (%)	MCC
1	78.57	80.65	79.66	0.5922
2	85.71	83.87	84.75	0.6951
3	92.86	80.65	86.44	0.7367
4	82.14	77.42	79.66	0.5949
5	85.71	77.42	81.36	0.6313
6	92.86	90.32	91.53	0.8308
7	89.29	96.77	93.22	0.8656
8	82.14	93.55	88.14	0.7649
9	82.14	77.42	79.66	0.5949
10	89.29	87.10	88.14	0.7629
Average	86.07±4.89	84.52±7.10	85.25±5.06	0.7069±0.10

To further evaluate the final classifier’s predictive power, an independent dataset was prepared by retrieving proteins that have a pairwise identity of <60% identity by CD-HIT and 25% ∼ 60% by BLAST with those in the whole training set. In consequence, 35 T3SPs and 86 non-T3 SPs were extracted as the testing samples. The prediction result shows that the predictor gives a good performance on this test set. The Sn, Sp and AUC are 94.29%, 91.86% and 0.9900, respectively (See Supplementary Table S3 and Figure 7). The detailed prediction result for each testing protein is shown in Supplementary Table S4.

### Experimental Validation of the Predictor

Finally, we used some candidate effectors to assess the practical feasibility of our predictor. Yang *et al.* [33] obtained 57 candidate effectors from rhizobial bacterial, 17 of these putative effectors have been verified as T3SPs by wet-bench experiments and the remaining 40 ones have not been experimentally confirmed. Thus, we used these proteins to test our predictor’s ability in identifying novel effectors. Removing the 9 sequences occurring in our dataset, 13 other experimentally confirmed effectors and 35 putative proteins were remained. Our method correctly predicted 11 from these 13 candidate T3SPs (Supplementary Table S5). In addition, Supplementary Table S6 presents the prediction results for the 35 rhizobial T3SPs that have not yet been confirmed and 28 of them are predicted as true positives by our method.

In addition, at the time of doing our calculations, Deng *et al.* have experimentally confirmed new T3SPs from enteropathogenic *Escherichia coli* (EPEC) and identified two novel effectors, C_0814/NleJ and LifA [83]. Using the 22 T3SPs confirmed by Deng *et al*. [83], our model was further tested. By excluding two proteins contained in our model and one with less than 100 amino acids, the remaining 19 T3SPs were predicted by our model (Supplementary Table S7). It shows that only 2 T3SPs were incorrectly predicted as other SPs and both of the novel effectors were correctly predicted. As for these new T3 members, we will add them to our model in the future, and we can expect that the performance would be further improved. Overall, the results demonstrate that if the sequence of a SP is obtained, novel T3SPs can be predicted effectively and efficiently using the predictor developed in this paper.

Meanwhile, these newly discovered effectors were also predicted by Effective T3, ANN, BPBAac and BEAN. The comparison results of our method with them are listed in Table 8. It further proves that our method does yields the strongest power in identifying novel effectors.

**Table 8:** The prediction results of different models on the newly discovered effectors.

Model	13 experimental effectorsby Yang et al. [33]	35 putative effectorsby Yang et al. [33]	19 new effectorsby Deng et al. [83]
RF (Our method)	10/12	28/35	17/19
BPBAac	10/12	6/35	9/19
EffectiveT3	5/12	2/35	13/19
ANN	10/12	16/35	17/19
BEAN	10/12	20/35	18/19

## Conclusion

Because previous methods have been successfully used to distinguish SPs from non-SPs, it is more essential to develop a computational method for effectively identifying T3SPs from non-T3 SPs. This paper introduces a new method for predicting novel T3SPs using position-specific residue conservation profiles. Based on the conservation profiles of the first 100 amino acids at the N-terminal region, 52 distinct position-specific residues were identified for T3SPs through the significant difference analysis by SAM. To our knowledge, this is the first attempt to identify distinct residues for T3SP prediction. Of the 52 distinct residues, the first 30 amino acid residues are all included, which is consistent with previous studies reporting that the secretion signal generally occurs within the first 30 residue positions and are most informative for T3SPs prediction. However, the remaining 22 positions span residues 30–100 have also proven by our method to contain important signal information for T3SP secretion because the physiologically significant translocation of many effectors also depends on the chaperone-binding residues or translocation signals that follow the secretion signal. The 62 selected optimal feature variables show that SA and SS are more important than AAC and PP, but some individual variables from AAC and PP also contribute to the mechanisms underlying T3SP secretion. Combining the information of four features, i.e., AAC, SS, RSA and PPs, that have been used in previous works for T3SPs representation, a permutation importance analysis was implemented for further feature optimisation, and 62 optimal feature variables were obtained. Moreover, feature importance analysis shows that SA and SS are more important than AAC and PP, but some individual variables from AAC and PP also contribute to the mechanisms underlying T3SP secretion.

Thus, based on the 52 distinct residues and 62 optimal feature variables, a RF predictor was constructed using an unbiased dataset of proteins with lower than 25% pairwise identity. The AUC is 0.9277 for the training set and 10-fold cross validation. When performed on the independent dataset, the model gives a high AUC of 0.9900.

Moreover, we compared our method with other four models. Our model consistently performed better than these methods. Lastly, we used other candidate effectors to test the model’s application, and the results also demonstrate that almost all T3SPs can be effectively identified using our model.

As the model was commonly constructed across 16 species, we believe that our method can be widely used for the efficient prediction of T3SPs in various bacterial species. Since T3SSs are central to the pathogenesis and virulence of many important Gram-negative bacterial pathogens, this work will help us to elucidate the secretion mechanism and further accelerate our understanding of pathogenic mechanisms and the development of potential therapeutics.

## Supporting Information

Table S1 **The statistical result of the secreted proteins data.**
(PDF)

Table S2 **Feature importance measure.** The discriminatory power of each feature was determined by calculating the importance value, with larger values indicating more relevant properties.
(PDF)

Table S3 **The prediction result of the final RF model on the independent dataset.**
(PDF)

Table S4 **The detailed prediction result for each testing protein with the predictive probability cut-off of 0.5 for T3SPs.**
(PDF)

Table S5 **The prediction result of experimentally confirmed secreted proteins with the predictive probability cut-off of 0.5 for T3SPs.**
(PDF)

Table S6 **Predicted novel secreted proteins in rhizobia that have not been confirmed experimentally with the predictive probability cut-off of 0.5 for T3SPs.**
(PDF)

Table S7 **The prediction results of the new T3SPs confirmed by Deng** ***et al.*** **(83).**
(PDF)
