# Supplementary material for: Effective Identification of Gram-Negative Bacterial Type III Secreted Effectors Using Position-Specific Residue Conservation Profiles
Source: PLoS One. 2013 Dec 31;8(12):e84439. doi: 10.1371/journal.pone.0084439 (PMC3877298; doi:10.1371/journal.pone.0084439)
Supplement: Table S1 — The statistical result of the secreted proteins data. (PDF) [file pone.0084439.s001.pdf]

Table S1. The statistical result of the secreted proteins data.

Secretory type	Number of sequences
Type I	87
Type II	186
Type III	662
Type IV	72
Type V	269
Type VI	10
Type VII	150
Type VIII	13
Total	1449
