# Supplementary material for: Effective Identification of Gram-Negative Bacterial Type III Secreted Effectors Using Position-Specific Residue Conservation Profiles
Source: PLoS One. 2013 Dec 31;8(12):e84439. doi: 10.1371/journal.pone.0084439 (PMC3877298; doi:10.1371/journal.pone.0084439)
Supplement: Table S2 — Feature importance measure. The discriminatory power of each feature was determined by calculating the importance value, with larger values indicating more relevant properties. (PDF) [file pone.0084439.s002.pdf]

Table S2. Feature importance measure. The discriminatory power of each feature was determined by calculating the importance value, with larger values indicating more relevant properties.

order	Feature	score	coverage ratio	order	Feature	score	coverage ratio
1	RSA18	12.50037	0.018276	118	SSH51	2.187703	0.003199
2	RSA17	12.47672	0.018242	119	SSE14	2.184221	0.003193
3	Hydro	11.83881	0.017309	120	SSC23	2.131799	0.003117
4	RSA13	11.43465	0.016718	121	SSC15	2.128444	0.003112
5	RSA16	9.688938	0.014166	122	SSE78	2.116347	0.003094
6	RSA15	9.542987	0.013952	123	SSE10	2.113245	0.00309
7	RSA04	9.238153	0.013507	124	SSC78	2.069273	0.003025
8	Npc	8.940048	0.013071	125	SSC22	1.992148	0.002913
9	RSA19	8.635378	0.012625	126	SSC24	1.986699	0.002905
10	RSA24	8.577556	0.012541	127	SSE36	1.963599	0.002871
11	RSA14	8.496652	0.012423	128	AAC(R)	1.910085	0.002793
12	SSC10	8.297427	0.012131	129	SSE30	1.902005	0.002781
13	SSC09	8.150919	0.011917	130	SSC29	1.900406	0.002778
14	SSE66	8.093057	0.011832	131	SSH22	1.845632	0.002698
15	RSA21	8.009703	0.011711	132	SSE09	1.834789	0.002683
16	SSC06	7.648721	0.011183	133	SSC76	1.809855	0.002646
17	RSA09	7.20957	0.010541	134	SSH27	1.799352	0.002631
18	RSA20	7.189312	0.010511	135	SSE52	1.784764	0.002609
19	SSC07	7.10113	0.010382	136	SSH24	1.774176	0.002594
20	SSE58	7.02992	0.010278	137	SSC48	1.763944	0.002579
21	RSA11	6.937114	0.010142	138	SSH23	1.726538	0.002524
22	RSA12	6.9079	0.0101	139	SSE20	1.689486	0.00247
23	RSA28	6.713684	0.009816	140	RSA39	1.67514	0.002449
24	SSH76	6.707353	0.009807	141	SSE50	1.654744	0.002419
25	SSH14	6.701334	0.009798	142	RSA71	1.626164	0.002378
26	RSA27	6.464362	0.009451	143	SSE26	1.604521	0.002346
27	RSA30	6.412951	0.009376	144	SSE28	1.553769	0.002272
28	SSC08	6.345432	0.009277	145	SSE03	1.545783	0.00226
29	AAC(E)	6.332586	0.009259	146	SSH25	1.532767	0.002241
30	SSH13	6.285567	0.00919	147	SSH97	1.517437	0.002219
31	SSE75	6.252446	0.009141	148	AAC(F)	1.51497	0.002215
32	RSA26	6.221109	0.009096	149	SSE96	1.454851	0.002127
33	RSA25	6.121136	0.008949	150	AAC(C)	1.454076	0.002126
34	Ins	6.093531	0.008909	151	SSE01	1.442234	0.002109
35	RSA29	6.034851	0.008823	152	SSE51	1.423141	0.002081
36	SSH78	5.967348	0.008725	153	SSE07	1.342402	0.001963
37	SSH75	5.849446	0.008552	154	SSE91	1.332524	0.001948
38	AAC(H)	5.60215	0.008191	155	SSC88	1.264829	0.001849
39	SSH66	5.528172	0.008082	156	SSC30	1.263246	0.001847
40	RSA22	5.520251	0.008071	157	SSE08	1.22434	0.00179

41	SSH15	5.375799	0.00786	158	RSA02	1.220483	0.001784
42	SSH94	5.313643	0.007769	159	SSE18	1.206026	0.001763
43	SSE71	5.270178	0.007705	160	RSA05	1.196977	0.00175
44	SSH68	5.213072	0.007622	161	SSE06	1.191472	0.001742
45	SSC11	5.145772	0.007523	162	SSE25	1.188903	0.001738
46	SSH71	5.055965	0.007392	163	SSH32	1.185341	0.001733
47	SSE12	4.912202	0.007182	164	SSH33	1.164097	0.001702
48	SSH58	4.885861	0.007143	165	SSC58	1.125796	0.001646
49	RSA10	4.83115	0.007063	166	SSC50	1.121432	0.00164
50	AAC(W)	4.679322	0.006841	167	SSH36	1.105506	0.001616
51	RSA23	4.650376	0.006799	168	SSE19	1.096961	0.001604
52	SSH12	4.585779	0.006705	169	SSE27	1.07182	0.001567
53	SSE13	4.544851	0.006645	170	SSH30	1.062404	0.001553
54	Ali	4.489038	0.006563	171	RSA01	1.041242	0.001522
55	SSC26	4.483211	0.006555	172	RSA36	1.025375	0.001499
56	SSC20	4.410194	0.006448	173	SSH28	0.94215	0.001377
57	SSE76	4.398171	0.00643	174	SSE97	0.940926	0.001376
58	RSA08	4.146082	0.006062	175	RSA35	0.933659	0.001365
59	SSC21	4.061282	0.005938	176	SSE15	0.888354	0.001299
60	RSA03	4.02736	0.005888	177	RSA51	0.882308	0.00129
61	SSC19	3.997723	0.005845	178	SSC33	0.8698	0.001272
62	SSC05	3.996422	0.005843	179	SSH35	0.84917	0.001242
63	SSH18	3.938828	0.005759	180	SSE35	0.833485	0.001219
64	AAC(Y)	3.908998	0.005715	181	SSC75	0.814841	0.001191
65	RSA06	3.88585	0.005681	182	SSE17	0.805407	0.001178
66	SSH03	3.833227	0.005604	183	SSC94	0.77491	0.001133
67	SSE94	3.742292	0.005471	184	RSA33	0.774626	0.001133
68	AAC(G)	3.711456	0.005426	185	SSE22	0.758862	0.001109
69	SSC12	3.683457	0.005385	186	SSH29	0.758718	0.001109
70	SSH48	3.67855	0.005378	187	AAC(P)	0.730484	0.001068
71	SSH08	3.605657	0.005272	188	SSE16	0.6979	0.00102
72	SSH17	3.58915	0.005248	189	SSC39	0.694819	0.001016
73	SSE11	3.578965	0.005233	190	RSA66	0.682125	0.000997
74	RSA78	3.556302	0.0052	191	RSA94	0.670823	0.000981
75	SSH50	3.540823	0.005177	192	RSA32	0.666498	0.000974
76	SSH04	3.53656	0.005171	193	SSC52	0.658186	0.000962
77	SSH07	3.535565	0.005169	194	RSA58	0.642941	0.00094
78	SSC27	3.494557	0.005109	195	SSE99	0.628945	0.00092
79	SSH96	3.470472	0.005074	196	SSH99	0.602753	0.000881
80	SSH19	3.408467	0.004983	197	SSC91	0.55279	0.000808
81	SSH52	3.370896	0.004928	198	SSC32	0.50472	0.000738
82	SSH16	3.303098	0.004829	199	AAC(L)	0.492132	0.00072
83	SSE68	3.284656	0.004802	200	SSE24	0.48633	0.000711
84	SSH11	3.242988	0.004741	201	RSA50	0.479863	0.000702

85	SSC25	3.201518	0.004681	202	SSC01	0.454024	0.000664
86	SSH09	3.1918	0.004667	203	SSE33	0.449089	0.000657
87	SSH10	3.158168	0.004617	204	SSC71	0.443392	0.000648
88	RSA07	3.091508	0.00452	205	SSE32	0.440712	0.000644
89	AAC(K)	3.011916	0.004404	206	SSC35	0.417567	0.000611
90	SSH05	2.874945	0.004203	207	RSA88	0.39704	0.00058
91	SSH20	2.840845	0.004153	208	SSC99	0.388634	0.000568
92	Nnc	2.816631	0.004118	209	pI	0.341138	0.000499
93	SSE48	2.793171	0.004084	210	SSE88	0.232865	0.00034
94	SSE04	2.728565	0.003989	211	AAC(T)	0.230398	0.000337
95	SSC17	2.724339	0.003983	212	SSC96	0.229375	0.000335
96	SSH06	2.663606	0.003894	213	AAC(Q)	0.221955	0.000325
97	SSC18	2.662152	0.003892	214	SSE23	0.218592	0.00032
98	SSC14	2.624227	0.003837	215	SSC51	0.211508	0.000309
99	SSC04	2.614711	0.003823	216	SSC97	0.125201	0.000183
100	SSH88	2.582176	0.003775	217	SSC02	0.097291	0.000142
101	SSH91	2.571108	0.003759	218	RSA96	0.081052	0.000119
102	SSH02	2.558985	0.003741	219	AAC(D)	0.05327	7.79E-05
103	AAC(S)	2.479211	0.003625	220	RSA99	0.049331	7.21E-05
104	SSC28	2.416009	0.003532	221	SSC68	0.043424	6.35E-05
105	AAC(A)	2.398518	0.003507	222	SSC66	0.005285	7.73E-06
106	SSC13	2.375922	0.003474	223	RSA68	-0.03109	——
107	SSH39	2.372692	0.003469	224	RSA75	-0.05248	——
108	SSE05	2.358333	0.003448	225	RSA97	-0.06225	——
109	SSE21	2.28615	0.003342	226	RSA48	-0.06448	——
110	SSH26	2.263325	0.003309	227	SSC36	-0.0723	——
111	SSE02	2.256964	0.0033	228	RSA91	-0.1858	——
112	SSE39	2.253738	0.003295	229	RSA52	-0.21303	——
113	SSH01	2.246445	0.003284	230	RSA76	-0.2137	——
114	SSC16	2.236727	0.00327	231	SSC03	-0.5291	——
115	SSH21	2.22142	0.003248	232	AAC(I)	-0.69302	——
116	AAC(V)	2.220641	0.003247	233	AAC(N)	-1.08187	——
117	SSE29	2.205237	0.003224	234	AAC(M)	-1.39946	——

AAC: amino acid composition; Three secondary structures: helix(SSH), beta strand (SSE) and coil (SSC); RSA: relative solvent accessibility; Six properties: theoretical pI, total number of negatively charged residues of Asp and Glu (Nnc) and positively charged residues of Arg and Lys (Npc), instability index (Ins), aliphatic index (Ali) and grand average hydrophobicity (Hydro).
