# Supplementary material for: Effective Identification of Gram-Negative Bacterial Type III Secreted Effectors Using Position-Specific Residue Conservation Profiles
Source: PLoS One. 2013 Dec 31;8(12):e84439. doi: 10.1371/journal.pone.0084439 (PMC3877298; doi:10.1371/journal.pone.0084439)
Supplement: Table S3 — The prediction result of the final RF model on the independent dataset. (PDF) [file pone.0084439.s003.pdf]

Table S3. The prediction result of the final RF model on the independent dataset.

No. of T3SPs	No. of non-T3 SPs	Sn (%)	Sp (%)	Acc (%)	MCC	AUC
35	86	94.29	91.86	92.56	0.8303	0.9900
