# Supplementary material for: Effective Identification of Gram-Negative Bacterial Type III Secreted Effectors Using Position-Specific Residue Conservation Profiles
Source: PLoS One. 2013 Dec 31;8(12):e84439. doi: 10.1371/journal.pone.0084439 (PMC3877298; doi:10.1371/journal.pone.0084439)
Supplement: Table S4 — The detailed prediction result for each testing protein with the predictive probability cut-off of 0.5 for T3SPs. (PDF) [file pone.0084439.s004.pdf]

Table S4. The detailed prediction result for each testing protein with the predictive probability cut-off of 0.5 for T3SPs.

Swiss-Prot ID	RF probability	T3SPs	Swiss-Prot ID	RF probability	T3SPs
A2A0X2	0.6032	yes	Q887C6	0.896	yes
A9MYP6	0.7284	yes	Q887D0	0.924	yes
B7V0T1	0.8188	yes	Q89T99	0.8204	yes
B8ZYE8	0.4972	no	Q8RP17	0.9924	yes
C6USR0	0.9288	yes	Q8ZQC8	0.8416	yes
D0ZRB2	0.6996	yes	Q9ANI0	0.7144	yes
D0ZVG2	0.706	yes	Q9FCY7	0.9292	yes
D2AJ72	0.732	yes	Q9FD10	0.8904	yes
D2TI21	0.8868	yes	Q9KH44	0.9212	yes
N0AC86	0.482	no	Q9L6W0	0.8968	yes
P18009	0.8976	yes	Q9WWH0	0.8976	yes
P18014	0.856	yes	Q9Z6N7	0.8524	yes
Q27RI1	0.8216	yes	Q9Z747	0.9212	yes
Q31SR9	0.676	yes	Q9Z753	0.884	yes
Q327E0	0.91	yes	Q9Z7D6	0.6208	yes
Q47279	0.6544	yes	Q9Z7N3	0.5764	yes
Q4ZMD6	0.8648	yes	Q9Z7Y1	0.7532	yes
Q840G7	0.9172	yes			
**Swiss-Prot ID**	**RF probability**	**non-T3SPs**	**Swiss-Prot ID**	**RF probability**	**non-T3SPs**
A7ZH66	0.7508	yes	Q07162	0.7084	yes
B4EYH3	0.9144	yes	Q09KJ5	0.6564	yes
B7LBY5	0.796	yes	Q0PAN9	0.6848	yes
B7MX50	0.6496	yes	Q0WA34	0.8952	yes
B7MX52	0.882	yes	Q1RDH0	0.9192	yes
B7NCM9	0.686	yes	Q3YL96	0.9612	yes
C8TQ66	0.8552	yes	Q46945	0.9204	yes
D2AJC0	0.7104	yes	Q54151	0.7684	yes
O32591	0.7204	yes	Q54450	0.8056	yes
O84419	0.5232	yes	Q54455	0.794	yes
O86164	0.786	yes	Q56806	0.8028	yes
O87657	0.9244	yes	Q59986	0.8052	yes
O87661	0.8028	yes	Q673F0	0.7672	yes
O88093	0.7932	yes	Q6D3F9	0.7036	yes
P04128	0.9324	yes	Q6D881	0.6288	yes
P05788	0.9156	yes	Q7B2V9	0.8544	yes
P07268	0.694	yes	Q7CL15	0.7048	yes

P08062	0.4124	no	Q7D2D9	0.2176	no
P08189	0.8804	yes	Q7N8E9	0.91	yes
P0C081	0.4416	no	Q7N8F0	0.942	yes
P0C1A2	0.8516	yes	Q7N8F1	0.8608	yes
P11312	0.8768	yes	Q8GH86	0.4352	no
P12730	0.9292	yes	Q8RTZ9	0.9064	yes
P13719	0.8756	yes	Q8VLQ5	0.8752	yes
P14212	0.8588	yes	Q8VT81	0.3716	no
P15320	0.9016	yes	Q92JF7	0.7412	yes
P15377	0.4552	no	Q93RN3	0.7292	yes
P15493	0.7324	yes	Q9ALK7	0.7728	yes
P16316	0.6148	yes	Q9ALL1	0.9552	yes
P16317	0.6388	yes	Q9EZE7	0.8108	yes
P16466	0.9	yes	Q9I747	0.644	yes
P21413	0.9248	yes	Q9PJY1	0.5692	yes
P24093	0.8644	yes	Q9PL47	0.7376	yes
P26876	0.7656	yes	Q9RB65	0.8652	yes
P33406	0.8668	yes	Q9RHT3	0.7228	yes
P35695	0.666	yes	Q9XB65	0.6316	yes
P43529	0.9496	yes	Q9XD84	0.8108	yes
P43660	0.9236	yes	Q9Z393	0.7972	yes
P44596	0.9328	yes	Q9Z398	0.8592	yes
P55128	0.5172	yes	Q9Z3A1	0.8932	yes
P62593	0.828	yes	Q9Z896	0.8316	yes
Q03011	0.9192	yes	Q9Z898	0.8688	yes
Q04681	0.9288	yes	Q9ZNI5	0.4768	no
