# Supplementary material for: Effective Identification of Gram-Negative Bacterial Type III Secreted Effectors Using Position-Specific Residue Conservation Profiles
Source: PLoS One. 2013 Dec 31;8(12):e84439. doi: 10.1371/journal.pone.0084439 (PMC3877298; doi:10.1371/journal.pone.0084439)
Supplement: Table S5 — The prediction result of experimentally confirmed secreted proteins with the predictive probability cut-off of 0.5 for T3SPs. (PDF) [file pone.0084439.s005.pdf]

Table S5. The prediction result of experimentally confirmed secreted proteins with the predictive probability cut-off of 0.5 for T3SPs.

Species	Effector	RF probability	T3SPs
Brady	Q89N83	0.6372	yes
Brady	Q89TP9	0.8444	yes
Meso	Q79UN8	0.93	yes
Meso	Q89BB8	0.946	yes
Meso	Q89TL5	0.9268	yes
Meso	Q989P6	0.7036	yes
Meso	Q989P8	0.9512	yes
Meso	Q9AMW4	0.8644	yes
Sino	P55711	0.3000	no
Sino	P55713	0.6876	yes
Sino	Q50EL2	0.8036	yes
Sino	Q5Y4S2	0.338	no
Sino	Q7BMF3	0.7004	yes
