# Supplementary material for: Effective Identification of Gram-Negative Bacterial Type III Secreted Effectors Using Position-Specific Residue Conservation Profiles
Source: PLoS One. 2013 Dec 31;8(12):e84439. doi: 10.1371/journal.pone.0084439 (PMC3877298; doi:10.1371/journal.pone.0084439)
Supplement: Table S6 — Predicted novel secreted proteins in rhizobia that have not been confirmed experimentally with the predictive probability cut-off of 0.5 for T3SPs. (PDF) [file pone.0084439.s006.pdf]

Table S6. Predicted novel secreted proteins in rhizobia that have not been confirmed experimentally with the predictive probability cut-off of 0.5 for T3SPs.

Gene ID	Swiss-Prot ID	Annotation	RF probability	T3SPs
bll0275	Q89XN3	hypothetical protein	0.8912	yes
bll1636	Q89TY7	hypothetical protein	0.826	yes
bll1648	Q89TX5	hypothetical protein	0.8116	yes
bll1796	Q89TQ4	hypothetical protein	0.6884	yes
bll1797	H7C7Y1	hypothetical protein	0.3768	no
bll1798	H7C6P6	hypothetical protein	0.824	yes
bll1804	H7C7U1	hypothetical protein	0.52	yes
bll1810	H7C6P5	hypothetical protein	0.8292	yes
bll1840	H7C7V0	hypothetical protein	0.8664	yes
bll1848	Q89TN8	hypothetical protein	0.6984	yes
bll1877	H7C6L9	hypothetical protein	0.8312	yes
bll5481	Q89J03	hypothetical protein	0.6028	yes
bll5622	Q89IL2	hypothetical protein	0.5208	yes
bll8244	Q89BB8	hypothetical protein	0.946	yes
blr0325	Q89XI9	hypothetical protein	0.7584	yes
blr0354	Q89XG0	hypothetical protein	0.6528	yes
blr1704	Q89TS9	hypothetical protein	0.9584	yes
blr1854	H7C7W8	hypothetical protein	0.9124	yes
blr1869	Q89TN2	hypothetical protein	0.8036	yes
blr5999	Q89HJ2	hypothetical protein	0.7824	yes
blr6167	Q89H28	hypothetical protein	0.2036	no
mll5027	Q98CS5	hypothetical protein	0.5464	yes
mlr0825	Q98LY1	hypothetical protein	0.8612	yes
mlr1025	Q98LH0	Transcriptional regulatory protein	0.1696	no
mlr3881	Q98F89	hypothetical protein	0.0396	no
mlr5875	Q98AS4	hypothetical protein	0.7204	yes
mlr6331	Q989Q3	hypothetical protein	0.7908	yes
mlr7808	Q984X0	hypothetical protein	0.9464	yes
Smed_0286	A6U665	hypothetical protein	0.2292	no
Smed_0887	A6U7W2	hypothetical protein	0.8476	yes
Smed_1170	A6U8P1	biotin-regulated protein	0.7412	yes
Smed_1171	A6U8P2	peptidase M23B	0.1732	no
Smed_4485	A6UI05	hypothetical protein	0.6444	yes
Smed_4857	A6UJ11	hypothetical protein	0.226	no
Smed_5711	A6ULA4	hypothetical protein	0.7268	yes
