# Supplementary material for: Effective Identification of Gram-Negative Bacterial Type III Secreted Effectors Using Position-Specific Residue Conservation Profiles
Source: PLoS One. 2013 Dec 31;8(12):e84439. doi: 10.1371/journal.pone.0084439 (PMC3877298; doi:10.1371/journal.pone.0084439)
Supplement: Table S7 — The prediction results of the new T3SPs confirmed by Deng et al. (83). (PDF) [file pone.0084439.s007.pdf]

Table S7. The prediction results of the new T3SPs confirmed by Deng *et al.* (83).

Protein name	Swiss-Prot ID	RF probability	T3SPs
LifA/Efa1*	B7UI23	0.986	yes
C_0814*	B7UMR0	0.5232	yes
NleB1	B7UI21	0.6272	yes
NleE	B7UI22	0.8688	yes
EspG2	B7UH72	0.518	yes
NleH2	B7ULW4	0.7656	yes
EspF	B7UM88	0.2472	no
Tir	B7UM99	0.9276	yes
Map	B7UMA0	0.7568	yes
EspH	B7UMA2	0.3132	no
EspG	B7UMC8	0.5836	yes
EspL	B7UI20	0.8784	yes
NleG	B7UNX2	0.6112	yes
NleB2	B7UNX3	0.8412	yes
NleC	B7UNX4	0.9728	yes
NleD	B7UNX6	0.7776	yes
NleA	B7UR60	0.9824	yes
NleF	B7UR63	0.7592	yes
EspB	Q05129	0.9396	yes

*denotes the novel effectors.
